# Identifying reliable indicators of fitness in polar bears

**DOI:** 10.1371/journal.pone.0237444

**Published:** 2020-08-19

**Authors:** Karyn D. Rode, Todd C. Atwood, Gregory W. Thiemann, Michelle St. Martin, Ryan R. Wilson, George M. Durner, Eric V. Regehr, Sandra L. Talbot, George K. Sage, Anthony M. Pagano, Kristin S. Simac

**Affiliations:** 1 U.S. Geological Survey, Alaska Science Center, Anchorage, Alaska, United States of America; 2 Faculty of Environmental Studies, York University, Toronto, Ontario, Canada; 3 U.S. Fish and Wildlife Service, Marine Mammals Management, Anchorage, Alaska, United States of America; 4 University of Washington, Polar Science Center, Seattle, Washington, United States of America; Cornell University, UNITED STATES

## Abstract

Animal structural body size and condition are often measured to evaluate individual health, identify responses to environmental change and food availability, and relate food availability to effects on reproduction and survival. A variety of condition metrics have been developed but relationships between these metrics and vital rates are rarely validated. Identifying an optimal approach to estimate the body condition of polar bears is needed to improve monitoring of their response to decline in sea ice habitat. Therefore, we examined relationships between several commonly used condition indices (CI), body mass, and size with female reproductive success and cub survival among polar bears (*Ursus maritimus*) measured in two subpopulations over three decades. To improve measurement and application of morphometrics and CIs, we also examined whether CIs are independent of age and structural size–an important assumption for monitoring temporal trends—and factors affecting measurement precision and accuracy. Maternal CIs and mass measured the fall prior to denning were related to cub production. Similarly, maternal CIs, mass, and length were related to the mass of cubs or yearlings that accompanied her. However, maternal body mass, but not CIs, measured in the spring was related to cub production and only maternal mass and length were related to the probability of cub survival. These results suggest that CIs may not be better indicators of fitness than body mass in part because CIs remove variation associated with body size that is important in affecting fitness. Further, CIs exhibited variable relationships with age for growing bears and were lower for longer bears despite body length being related to cub survival and female reproductive success. These results are consistent with findings from other species indicating that body mass is a useful metric to link environmental conditions and population dynamics.

## Introduction

Measures of animal body size and condition can be useful indicators of population responses to environmental change, linking nutritional intake to effects on reproduction and survival [1–7]. Body condition is a term that is often used to reflect an animal’s current energy reserves (i.e., the amount of energy stored in tissues) [8–10] available to support reproduction, early survival of young, and survival during periods of food scarcity [11, 12]. Declines in body condition and changes in growth patterns can be indicative of reduced food intake either due to competition resulting from increased population density [13], declines in food availability and quality [4, 14] or increased energetic costs [15]. Because measuring changes in population size and vital rates is challenging for many wildlife populations [16], temporal trends in body condition can be a useful indicator to infer population status, identify nutritional or energetic mechanisms that may be limiting populations [17, 18], and serve as an early warning sign of population change [4, 5, 14].

Condition indices (CIs) are often generated to provide a single metric that can be used to assess and compare the energy reserves of individuals across individuals of varying age or body size (i.e., that the metric would accurately assess relative condition such that an animal of larger body size did not have a higher CI) [10, 19–21]. This then allows CIs (i.e., condition metrics that standardize for structural size) of individuals of varying age and size to be analyzed together to examine temporal trends and relationships with environmental and other parameters [19]. However, a variety of approaches have been used to generate CIs with mixed success relating to direct measures of energy reserves (e.g., quantified body composition) or to reproductive and survival outcomes [9]. Residuals from a regression of body mass relative to body length, the latter used as a proxy for structural size, have commonly been used as CIs. But this approach does not always adequately account for length variation [21], can result in poor prediction of body fat content [22, 23] which is the primary energy storage for most mammals, and be prone to significant error when correlated with length [24]. In some cases, body mass has performed similar to or better than CIs that standardized for structural size in relating to direct measures of available energy reserves [birds: 11, 25; small mammals: 26; large mammals: 27, 28], reproductive success [29, 30], and offspring survival [31]. In a review of measures used to assess body condition (i.e. not just CIs but also approaches that do not standardize for structural size), the authors concluded that there was no consensus on appropriate condition metrics and that often the condition metrics applied within a discipline are those used in previous studies [21]. Because energy reserves and the degree to which they are used to support survival and reproduction vary widely across species as a result of differences in life history traits, the most meaningful condition metrics may be taxon-specific. For example, percent body fat varies from <5% in some small mammals [26] to over 49% in brown (*Ursus arctos*) and polar bears (*U*. *maritimus*) [32, 33]. Additionally, species that rely on energy reserves to support pregnancy or lactation (i.e., capital breeders) demonstrate stronger relationships between condition and fitness outcomes [34] than those that rely on energy obtained from actively foraging during lactation (i.e., income breeders) [35]. Most CIs are validated by relating the index to a measure of an animal’s energy reserves, including either fat alone or combined energy from fat and muscle [10, 19, 20, 22]. The assumption is that energy reserves will contribute to reproductive and survival outcomes, but few CIs are validated as direct indicators of fitness [36].

Identifying an optimal approach to estimate the body condition of polar bears is currently needed to improve monitoring of their response to the rapid and significant decline in their sea-ice habitat [5, 37, 38]. Polar bears feed primarily on seals that they access from the sea ice, hence, declines in sea-ice extent can reduce their ability to access prey [14, 39] resulting in potential direct effects on their condition. Declines in body condition have been observed in polar bears concurrent to declines in their Arctic sea ice habitat [5, 16, 37, 40]. For polar bears and other ursids in temperate regions, energy reserves are critical to reproduction because females produce young in winter dens while fasting [12, 41, 42]. Although some lean body mass is catabolized during periods of food scarcity, stored fat is the primary source of energy supporting winter survival and reproduction. In polar bears, only pregnant females den and they derive 93% of energy from fat while denning [41]. Similarly, non-lactating, hibernating brown bears (males and females) derive nearly all energy from fat reserves [43]. Condition of pregnant female bears has been found to dictate the likelihood of producing offspring [41, 44, 45], date of parturition and cub growth [42, 44], litter size [44] and cub mass and survival [46, 47]. Further, female condition at den entry can affect the timing of birth in the den [42] and thereby, the size of a cub when it exits the den and its survival during the year after den emergence [46, 47]. Thus, female body condition in bears can be an important determinant of reproduction and cub survival.

A wide variety of measures have been applied under varying ecological scenarios (e.g. actively feeding versus fasting bears, fall versus spring measures) to quantify condition in polar bears. These include a body mass index (BMI) in which mass is divided by the square of body length [20], a body condition index (BCI) based on residuals of the relationship between mass and length [19, 37, 48], and energy density based on equations using previously published measures of body composition [10], all of which incorporate measures of body mass and length. A subjective, categorical index of condition ranging from 1–5 [“fatness index” (FI); 20] and percent lipid content in adipose tissue biopsies [38] have also been used to assess body condition without requiring body measurements. These CIs (BMI, BCI, energy density and FI) have been used to assess long-term trends in body condition with the implicit assumption that they represent responses to environmental variation and are indicative of the likelihood of reproductive success and survival [e.g., 5, 37, 48, 49]. Further, they are intended to be comparable across individual bears regardless of sex, age or reproductive state [19] such that individuals of different sex and age classes can be combined to examine temporal trends [e.g., 37, 38]. The validity of some CIs has been assessed by relating them to direct measures of body composition (i.e., sacrificing of animals and quantifying tissue amounts and tissue energy content) [19, 50] or to other CIs [10, 20, 38], but limited data are available to confirm whether these metrics are related to foraging success, reproduction, or survival in polar bears [15]. BCI, BMI, and energy density have in common that they attempt to quantify mass or energy reserves in proportion to structural size. However, larger structural size alone may be advantageous for polar bears in part because large-bodied prey can be more effectively captured by larger predators [51, 52]. Further, polar bears are sexually size dimorphic and larger males have an advantage in competition for mates [53–55]. Thus, structural size may be an important factor affecting fitness in polar bears.

Most body condition metrics are based on body measurement data, but measurement protocols for polar bears are not standardized. Length has been measured as a straight line distance between the tip of the nose and either the base of the tail [5, 48, 56] or the tip of the tail [5, 19, 38, 57] with a measuring tape above the bear in sternal recumbency or along the body contour with the bear on its side [USGS, unpub data] or in sternal recumbency [38, 50]. Similarly, body mass of polar bears is measured directly [5, 15, 19] or calculated from measures of girth and length [38, 48, 49, 56–58] or girth alone [59]. Calculated mass has been suggested as a preferred approach because polar bears consume large meals [57] and therefore can carry significant mass in ingesta [60]. Hence, there is substantial variation in measurements and CIs used to monitor polar bear body condition with no consensus on an optimal approach.

Identifying accurate and precise measures to assess condition of young, growing animals may be a particularly important component of population monitoring. Population dynamics of large mammals are most sensitive to adult female survival [61], but the survival rates of young, growing animals are often more variable than those of adults [62–65] and are among the first vital rates to decline as a result of environmental change [5, 62, 66, 67]. In the southern Beaufort Sea where polar bears have experienced substantial declines in sea ice habitat and exhibited declines in body condition and prey capture efficiencies [5, 68], a decline in population abundance was associated with near 0 cub survival between 2003 and 2007 [62]. Thus, as sea ice loss occurs, survival of young polar bears may be an important mechanism by which population abundance is affected [62, 69]. Although reproductive and survival rates can be directly measured for some bear populations, accurate estimation of reproduction and survival rates in polar bears generally requires intensive study over 3–10 year periods (depending on recapture rates and accessibility of individuals in the population) and can be characterized by large uncertainty [62, 70–72]. Thus, identifying additional measurable parameters that can be collected on a more frequent basis and accurately reflect reproductive potential and juvenile survival are important to support population monitoring [62, 72, 73].

Here, we use three decades of measurement data from over 3000 polar bears captured from the Chukchi Sea (CS) and Southern Beaufort Sea (SB) subpopulations to identify measurements that can be collected with precision and accuracy and that predict female reproductive success and cub survival. Specifically, our objectives were to: (i) examine relationships between direct body measurements (i.e., girth, length), metrics that combine structural size and condition (e.g., body mass), and CIs (that standardize across bears varying in structural size) and indicators of female reproductive success, including cub production (i.e., whether or not a female produced cubs), litter mass, and cub survival, (ii) examine relationships between cub size and condition and survival for the year after den emergence, and (iii) examine factors that affect the application of CIs and other morphometrics as indicators of fitness. These include variation in CIs with length and age and the precision and accuracy of body measurements, including inter-observer variation and effects of ingesta (i.e., stomach contents) on body mass.

## Materials and methods

### Morphometric measures and CIs

**Fig 1:**
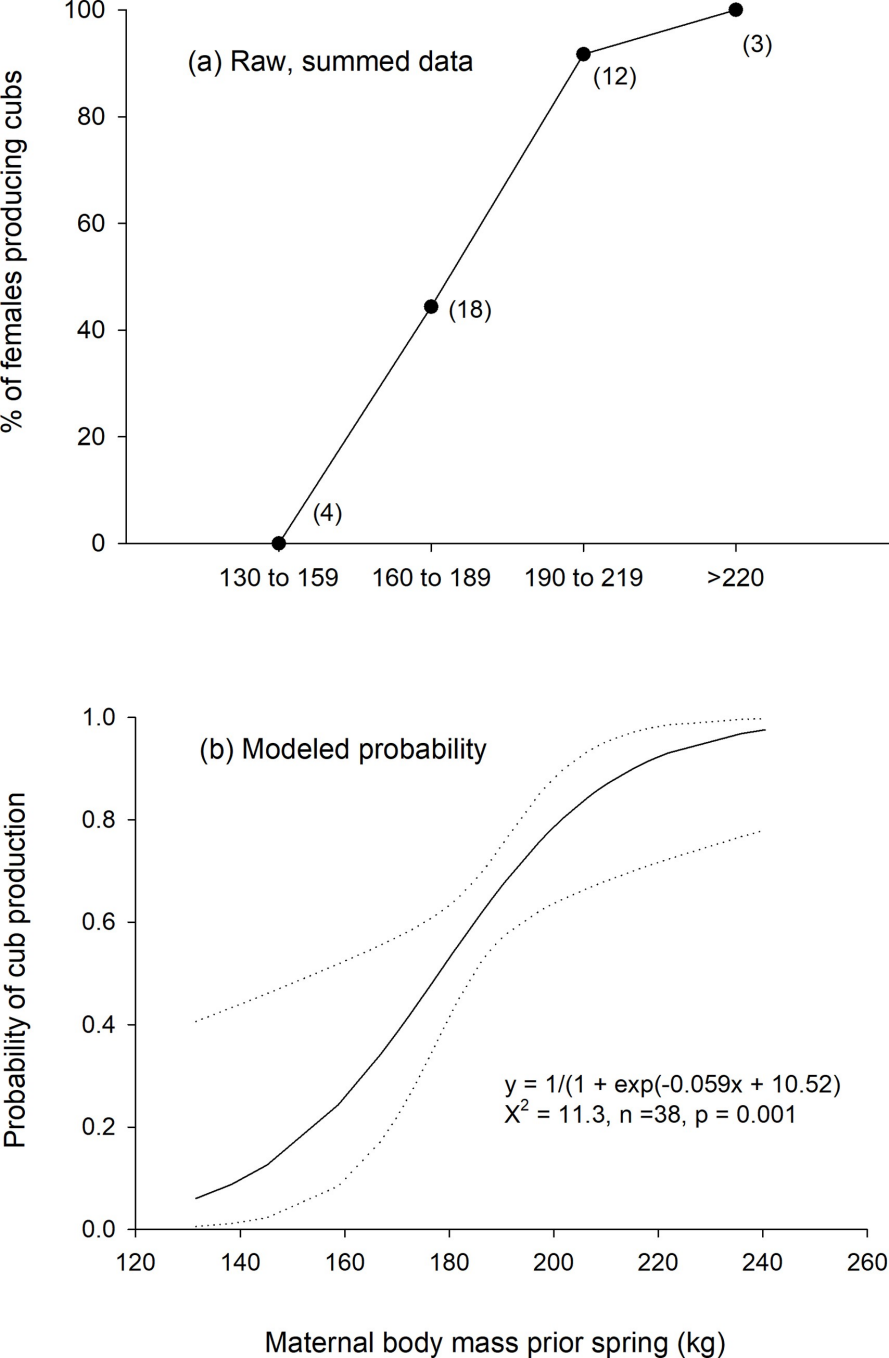
Plot of the percent of females age 5 and older observed with spring cubs (year t + 1) grouped by their body mass measured the prior spring (year t)(a) and the modeled probability (with 95% confidence intervals as dashed lines) of cub production relative to maternal body mass from a logistic regression (b).

Measurement data described in Table 1 were obtained from polar bears captured off the Alaskan coast of the SB and throughout the Russian and American portions of the CS between 1971 and 2016 (see Fig 1 in 68 for sampling locations). These included 2,512 captures of all sex and age classes in the SB (1,961 in March-May (spring) of 1981–1994 and 1996–2016 and 551 in Sept-Nov (fall) of 1983–1986, 1989, 1993–1994, 1996–2001, and 2008–2009), 468 captures of polar bears in the Alaska portion of the CS (in March-April of 2008–2011, 2013, and 2015–2017), and 336 captures March to early May targeting adult females with first year cubs within the Russian and Alaskan portion of the CS 1986 to 1996. Only eight adult males were captured in the latter data set. First year cubs were rarely captured (n = 3) in the CS 2008–2017 because females den primarily in Russia and do not move into Alaskan waters until later in the spring [74]. Therefore, no cub data from the CS 2008–2017 were used in analyses. Cubs remain with their mothers for approximately 2.5 years in the two study subpopulations. Thus, females can be observed with first-year cubs that were born in January, second-year cubs (yearlings), or third-year cubs (two-year-olds). Polar bears were located using a helicopter or fixed-wing aircraft and immobilized with a rapid-injection dart containing zolazepam-tiletamine (Telazol^®^ or Zoletil^®^) fired from a helicopter [75] 1987–2017 or Sernylan (phencyclidine hydrochloride) or M-99 (etorphine) prior to 1987. Locations of capture areas were primarily on the sea ice with the exception of some fall captures on land [68]. Studies were conducted under U.S. Fish and Wildlife Service research permits MA 690038 and 046081and followed protocols approved by the U.S. Geological Survey’s Alaska Science Center and U.S. Fish and Wildlife Service Alaska Regional Office Animal Care and Use Committees.

**Table 1:** Measurements collected from Chukchi Sea and Beaufort Sea polar bears.

Measurement or sample name	Description
Body mass (kg)	Measure of mass using a load cell, tripod, and chain hoist.
Age (years)	Measured from annuli in an extracted vestigial premolar or via capture as dependent young in which first and second year cubs were differentiated based on body size and dentition.
Body length (cm)	Throughout the manuscript body length refers to a “straight line body length” (SLBL) measure unless otherwise stated. SLBL is described below.
Straight line body length (length)^1^ (cm)	Measure of length quantified as the straight line distance from the tip of the nose to either the end of the last tail vertebrae (for bears caught after 2001) or to the base of the tail (for bears caught prior to 2002) using a measuring tape held horizontally several cm above the bear in sternal recumbency avoiding variation in the body contour.
Total body length (cm)	Measure of length quantified as the distance from a bear’s nose to the tip of the last tail vertebrae following the body contour with the bear lying on its side.
Tail length (cm)	Measure from the base of the tail to the last tail vertebrae. Mean tail lengths were used to standardize SLBL measures by adding mean tail length for measures collected prior to 2002 [5].
Skull width (cm)	Measure of the zygomatic width of the skull using calipers.
Girth (cm)	Measurement of the circumference of the abdomen immediately behind the forelimbs using a rope tightened to matte the fur but not indent the skin [49].
Fatness index (no units)	A categorical indicator of body condition ranging as an integer from 1 (skinny) to 5 (fat) that is assigned based on definitions in [20].

^1^ This is the most commonly collected and applied length measure for polar bears [5, 19, 38, 49, 56–58]

In addition to measurements, each independent bear was assigned a subjective, categorical “fatness index” ranging from 1 to 5 as described in [20]. Because FIs of 1 (skinny) and 5 (fat) were uncommon (e.g. among 386 adult females caught in the SB, ten were scored as 1 and one was scored as 5), we combined 1s and 2s and 4s and 5s resulting in three categories (1 & 2, 3, 4 & 5) [consistent with 76]. FIs were not consistently assigned to dependent young and therefore were only included in analyses assessing condition of independent bears. Body mass (referred to as “mass” or “measured body mass”) and straight line body length (referred to as “length” and defined in Table 1) measures were used to calculate the three measurement-based CIs commonly applied to polar bears: BMI [5, 14, 20], BCI [37, 48, 77], and energy density [10, 38, 77, 78]. BCI was calculated separately for males and females in each subpopulation [37]. Equations for estimating energy density were not available for subadult females [10]. In addition to calculating CIs, we calculated storage energy [10] that estimates energy reserves. Like body mass, storage energy is not scaled in proportion to total body size.

Bears in this study were weighed in a net suspended from a hanging scale but it is common practice to calculate polar bear body mass from equations using girth and length measures [49, 56, 58]. Calculating body mass has been suggested as a way to minimize effects of ingesta [79] which can add significant weight for polar bears [58, 60] and therefore affect measured body mass. Further, calculating body mass from measurements avoids the limitations of carrying the additional heavyweight tripod, chain-hoist and net required to weigh bears, particularly when most bears are captured via helicopter which have weight limits for hauling gear. However, because calculated body mass in CIs results in cumulative bias (i.e., additive error) [19, 37], we calculated all CIs using measured body mass. We did, however, investigate relationships between calculated body mass and fitness metrics (as described below), along with the direct measurements (length, skull width, body mass) and CIs since calculated body mass is commonly used in population assessments [46, 47, 49, 58, 80]. Equations for calculating body mass from girth and length measures were developed separately for males and females and for bears captured before and after the year 2000 in the SB due to changes in sea ice conditions, feeding behavior, and bear body condition [5, 62, 68] consistent with recommendations that equations for calculating body mass be periodically updated [49, 56, 62]. We note in the discussion the potential error introduced by using different equations to calculate body mass and examining long-term trends.

For all analyses, bears were separated into the following sex and age classes: males age 2–10 (“growing” males), females age 2–5 (“growing females”), males age ≥11 years old (“adult males”), females age ≥ 6 years (“adult females”), and yearlings. Age classes were defined based on growth curves where independent females age 2–5 and males age 2–10 exhibit growth in structural size (i.e., skull width and length) with a steep slope between age and mass or length and females ≥6 years old and males ≥11 years old have a reduced or negligible slope between age and mass and length [5, 77]. Analyses of yearling body measurements and CIs included both sexes but sex was included as a fixed effect to account for sexual dimorphism that has been observed in this age group [46]. Older bears were analyzed separately for the two sexes due to differences in the age in which they reach asymptotic body size.

### Identifying body measurements and CIs related to female reproductive success and cub survival

Table 2 describes the logistic and linear regression models and associated covariates included in candidate models to examine potential relationships between CIs, body measures, or estimates of body mass and storage energy with indicators of female reproductive success and cub survival. Three types of metrics of bear size and condition were examined: 1. Measures of size (body length), 2. Measures that combine size and fatness (measured and calculated body mass, storage energy, skull width and girth), and 3. Condition indices, including BCI, BMI, energy density, and FI. Collectively we refer to types 2 and 3 as “condition measures” because they both incorporate potential seasonal variation in energy reserves whereas 1 should be independent of these effects. Because skull width was measured on live bears, it can represent both size and condition [5]. Indices of female reproductive success were assessed via 3 measures: cub production (a binomial measure of whether or not a female was observed with cubs after denning) determined from observations of females in the spring of year *t* + 1 after den emergence and related to maternal condition measured the prior spring or fall of year *t*; spring litter mass at time *t* (accounting for potential litter size effects) related to maternal condition measured at time *t*, and survival of first-year cubs (referred to as “cubs”) from the spring of year *t* to the spring of year *t* + 1 related to maternal condition in the spring of year *t*. Females were observed directly during capture with the exception that observation from aircraft during radiotracking were sometimes used to determine whether they were accompanied by cubs or yearlings. Cub production was determined for females that were captured alone or with two-year olds in the spring prior to denning (i.e. because females are often in the process of weaning two-year olds in the spring and subsequently mate and produce cubs the following spring of year t + 1). Females with yearlings in the spring are unlikely to mate and therefore were excluded as candidates to contribute to cub production the following spring. Although we refer to this measure as “cub production” it integrates whether or not a female bred the prior spring and produced cubs and whether or not they survived to be observed during the spring capture season following denning (note below that capture date was included in as a covariate in models of cub production). We also examined whether maternal body measures and CIs were related to the litter mass of cubs or yearlings that accompanied her at the time of capture (i.e., maternal condition and litter mass measured at year t) where data were available for females from both the CS and SB.

**Table 2:** The structure and covariates included in candidate models used to identify body size and condition measures that were related to indices of female reproductive success and cub survival.

Fitness measure	Model	Dependent Variable	Related to	Covariates
FEMALE REPRODUCTIVE SUCCESS				
Cub production^1^	Logistic regression	0 = did not produce cubs; 1 = produced cubs in year *t* + 1	Maternal size or condition ***during the prior spring*** or fall (year *t*)	Cub capture date, age
Litter mass^1^	Linear regression (general linear model)	Combined mass of all cubs in a litter. Cubs and yearlings were run in separate models. (year *t* + 1)	Maternal size, or condition ***during the prior*** spring or fall (year t)	Cub capture date, litter size, age
Litter mass^2^	Linear regression (general linear model)	Combined mass of all cubs in a litter. Cubs and yearlings were run in separate models. (year t)	Maternal size, or condition ***when accompanied*** with the litter (year t)	Cub capture date, litter size, subpopulation, age
Cub survival^1^	Logistic regression	0 = cub did not survive 1 = cub survived from spring of year t to spring of year t + 1	Maternal size or condition in the spring after den emergence (year t)	Maternal ID, cub capture date, age
CUB SURVIVAL				
Cub survival^1^	Logistic regression	0 = cub did not survive 1 = cub survived from spring of year t to spring of year t + 1	Cub size or condition (year t)	Cub capture date

For each model the following size and condition measures were entered separately: body length, girth, skull width, mass, calculated mass, BCI, BMI, energy density, and fatness index. These factors were included in separate models as either linear or quadratic terms to allow for potential non-linear relationships. Female reproductive success was quantified via 3 different measures: cub production (whether she produced cubs that survived to be captured the spring following denning), the mass of the litter she produced if she produced cubs (litter mass), and cub survival. Cub production was determined via observations of females in the spring post-denning. Capture date was included in candidate models of cub production and cub survival due to the potential for bears observed later to have more time for cub mortality to occur. Litter mass models were run separately for cubs and yearlings. Maternal ID was included as a random effect in models of cub survival to account for repeated measures of females that were initially observed with more than one cub. Age was included as a linear or quadratic term in candidate models with and without size and condition measures.

^1^ Data available only for Southern Beaufort Sea bears

^2^ Data available for Southern Beaufort Sea and Chukchi Sea bears

We related measures of females collected in the spring and the fall of year t to cub production and litter mass measured in the spring of year t + 1 because polar bears cannot be consistently captured during either season for all subpopulations. Although polar bears summer onshore in some subpopulations, in other areas the majority of bears summer on the sea ice far from shore where they are inaccessible for capture. Because pregnant females must obtain sufficient energy reserves to meet the demands of gestation and lactation while fasting in the den, differences in female body condition in the fall are likely to be indicative of differences in reproductive success. Alternatively, females measured in the spring may change substantially in condition by the fall depending on movement patterns and predation success resulting in weaker relationships with reproductive outcomes a year later. Thus, we sought to determine whether measures collected during these two seasons are predictive of reproductive outcomes.

Survival of cubs was determined for mothers who were accompanied by cubs after denning and were recaptured the following spring. Because some females had more than one cub in which survival was determined, maternal ID (i.e. bear number) was included as a random effect in models of cub survival to account for repeated measures of females that were initially observed with more than one cub. We assumed that a cub that had been captured initially with a female had died if it did not accompany her at recapture a year later since females in our study area rarely wean cubs within the first 1.5 years. All cubs received lip tattoos which allowed identification of individual survival of cubs within litters. This approach of relating cub survival to maternal condition differs from population-estimates of cub survival which require incorporation of variability in detectability and maternal survival. Thus, the absolute values of cub survival reported here should not be equated to population-level estimates. Rather we assumed that females identified with cubs that did or did not survive exhibited representative body condition for females with those reproductive outcomes. Because recapture rates in the CS are low, sufficient data for relating female spring and fall condition to reproductive outcomes the following spring were only available for SB females.

To assess whether size and condition measures were related to indices of female reproductive success and cub survival, we entered size or condition measures individually in linear or logistic models. Prior to examining these relationships, we examined whether other factors, such as subpopulation, capture date or maternal age (listed in Table 2), influenced reproductive success and cub survival and therefore should be included in the models with the size or condition measure. Factors, including body size and condition measures, were considered influential in affecting female reproductive success or cub survival when the 95% confidence interval (CI) of the size or condition metric’s coefficient (β) in the model did not overlap zero [81–83] and p ≤ 0.05. Size and condition measures were entered in separate models as either linear or quadratic terms (i.e. to allow for non-linear relationships with the reproductive index). Including more than one size or condition measure simultaneously would violate assumptions of non-collinearity. Our primary objective was to identify the size and condition measures that were related to fitness indices. Maternal age was included in individual models without size or condition metrics to determine the degree to which age alone, due to its relationship with bear size and mass, might affect fitness.

Because FI is a categorical variable, we provide β with 95%CIs comparing between categories where β_1–3_ indicates a comparison between scores 1 and 3 and β_2–3_ = indicates a comparison between scores 2 and 3 to evaluate whether differences between categories were related to fitness indices. When no covariates were influential resulting in FI being related to fitness indices with no other variables, we ran an ANOVA with a Bonferroni post-hoc test to compare fitness indices between bears of different FIs.

Because analysis of long-term trends in female body condition requires understanding how condition may vary with reproductive status, we compared the condition between females accompanied by cubs, yearlings, or two-year olds and lone females. An ANCOVA was used to compare across females of differing reproductive status (as a categorical fixed effect) with capture date as a covariate and the size or condition metric as the dependent variable.

### Variation in CIs associated with age and length

An important assumption in the application of many CIs is that they can be compared across individuals that vary in age or structural body size [19, 37, 48]. We examined relationships between individual CIs (dependent variables) and age and body length (independent variables) using linear regression (i.e. separate regressions to examine the relationship between each CI with age or body length). Because relationships between age and body size have been shown to differ between bears in the two subpopulations [77], we included population as a factor in all regressions. We also examined relationships between age and body length with direct body measures (skull width, girth, and body mass) within the defined sex and age groups to better understand how morphometric variation may contribute to the patterns we observed in CIs and with two calculated measures commonly used to represent body mass (calculated body mass [55, 65, 66] and storage energy) [10].

### Precision and accuracy of body measurements

Body size measurements can include error resulting from different observers or positioning of the bear [80]. Straight-line length, a common metric, requires measuring a bear while it is sternally recumbent. The tape is stretched above the bear, from its nose to the base of its tail, but does not follow contours of the body. An alternative metric, total-body length, follows the body contour while the bear lies on its side. The tape is positioned at the nose and tail. We evaluated variation in straight-line and total-body length measurements for SB and CS bears captured two times or more after 2002, when length was measured consistently to the tip of the tail. Analyses were restricted to male and female bears (separately) age ≥11 years, because these individuals should have been fully grown. To determine whether length measures were larger on subsequent captures which might suggest continued growth in length, we conducted paired t-tests of length measures between the first and second capture.

We compared size and condition measures between bears identified as having fed or not fed just prior to capture to determine if ingesta may affect body measurements and CIs [61, 65]. Data were used from CS polar bears sampled 2009–2017 where estimates of gut ingesta had been recorded. Quantity of ingesta (gutfill) was estimated based on gut palpation, direct observations of feeding prior to capture, and fecal sampling. “Full” bears were identified based on tight, extended bellies and/or observations of feeding on a carcass just prior to capture and “empty” bears were those that were not observed feeding prior to capture, had bellies that could be palpated with no apparent sounds or movement of a recent meal, did not defecate during capture, and had no fecal material within their rectum. Bears that were identified as partially full and not likely to either be completely empty or full were excluded from analysis to minimize inclusion of bears in which gut fill was less certain. ANCOVAs were used to compare size and condition measures between bears that were identified with empty guts versus those identified as full. Capture date and age effects were included in ANCOVAs for growing and adult males and females if *p* ≤ 0.05. ANCOVAs for yearlings included a sex effect and capture date.

Because body mass is commonly calculated in polar bears from girth and length measures, we examined differences between calculated and measured body mass collected from the same individuals for bears that were identified as having not fed prior to capture (i.e., whose body mass would not be affected by ingesta identified only in CS bears sampled 2009–2017). We similarly examined this relationship for bears that fed (i.e., to determine the potential bias associated with ingesta) to determine the difference between calculated and measured body mass for bears whose measured body mass was affected by the weight of ingesta. We used ANCOVA with body mass as the dependent variable, calculated mass as a linear covariate, and fed or not fed as a fixed, binomial effect. We quantified the difference between calculated mass and measured mass using the absolute percent difference (i.e., independent of the directionality of the difference) as well as using the percent difference between the two measures–the latter of which assesses directional bias (i.e., whether calculated mass overestimates or underestimates measured mass).

All analyses were conducted in IBM SPSS statistical software Version 26.0.0.0.

## Results

### Body measurements and CIs related to female reproductive success

**Fig 2:**
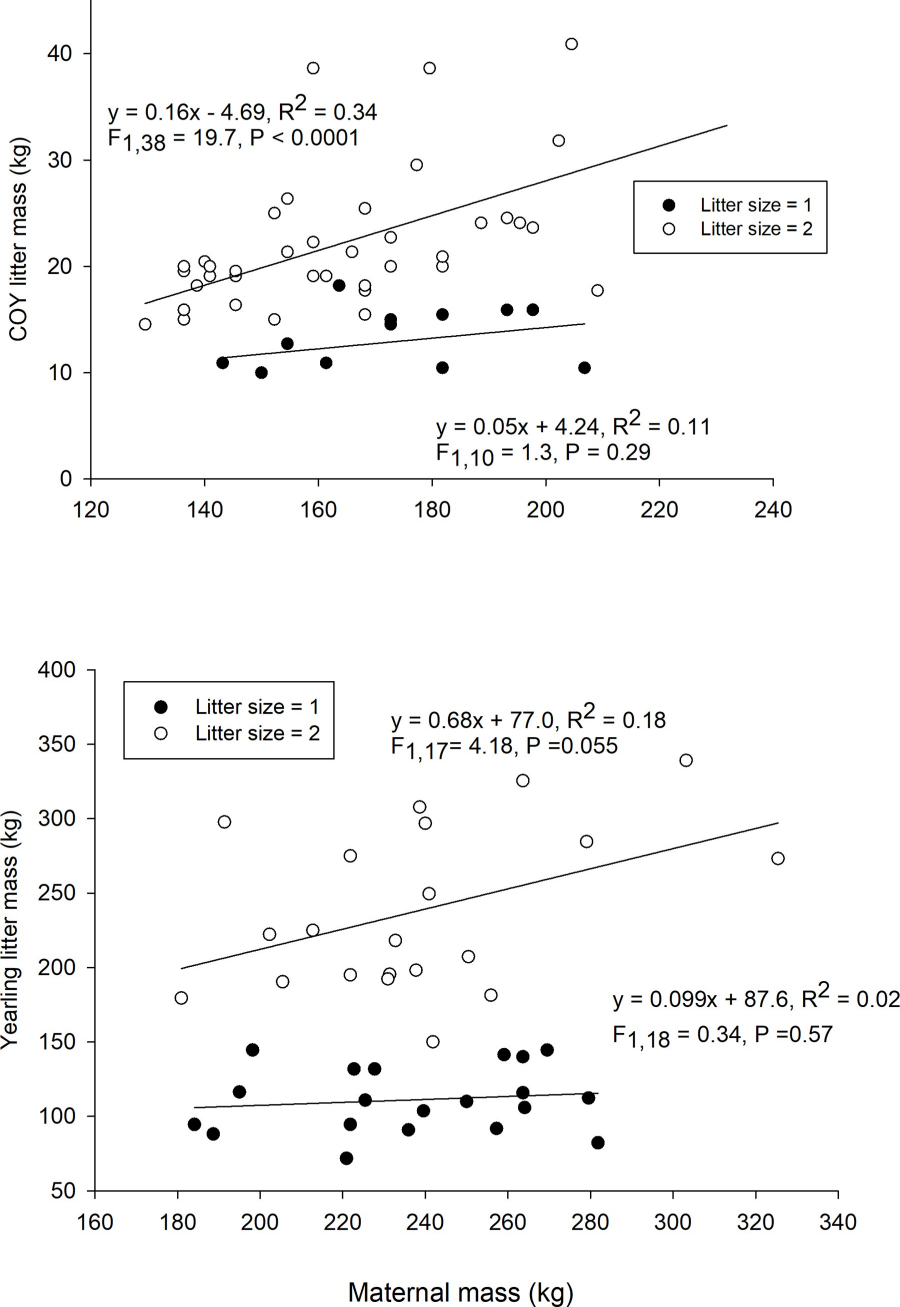
Relationships between maternal mass and the mass of first year cubs (i.e., COY) and yearlings in one and two cub litters of adult female polar bears captured in the spring in the Chukchi Sea 1986–1994 (a) and yearlings captured in the Chukchi Sea 2008–2017 (b).

Adult females with higher body mass in the spring of year t had a higher probability of being observed with cubs in the spring of year t + 1. Spring body mass was linearly (log-likelihood = -16.9, β = 0.06, 95% CI = 0.02–0.10, n = 37, p = 0.008; Fig 2; S1 Table) and non-linearly (quadratic: log-likelihood = -17.6, β = 0.00012, 95% CI = 0.00002–0.00015, n = 37, p = 0.014; S1 Table) related to cub production. Spring calculated body mass was also linearly related to cub production (log-likelihood = -21.8, β = 0.03; 95% CI = 0.0004–0.06, n = 36, p = 0.05; S1 Table). Cub production (in year t + 1) was not related to CIs, storage energy, girth, or length of spring-caught females (in year t) (S1 Table) nor to capture date. Similarly, age was not related to cub production when included in models with or without size and condition metrics. The log-likelihood was lowest for each condition metric when no other covariates were included in the models and when the linear term was used in the model (S1 Table). Cub production did not differ between females with different FI scores in the spring (β_1–3_ = 1.4, 95% CI = -0.6–3.4, p = 0.18; β_2–3_ = -0.5, 95% CI = -2.4–1.4, p = 0.58).

Cub production (at spring t + 1) was related to maternal energy density, body mass, BMI, BCI, and storage energy measured in the fall of year t (S1 Table). The model including energy density and no other variables had the lowest log-likelihood (log-likelihood = -5.0, β = 0.32, 95% CI = 0.001–0.650, n = 20, p = 0.05) (S2 Table). Neither capture date nor age were related to cub production and therefore, were excluded from all models. Cub production did not differ between females with different FI scores in the fall (β_1–3_ = 1.8, 95%CI = -1.7–5.3, p = 0.31; β_2–3_ = 0.41, 95% CI = -2.2–3.0, p = 0.76). The lowest mass of a female that was later observed with cubs was 167 kg in the spring and 248 kg in the fall. Females that produced cubs lost on average 118.7 ± 8.4 kg (mean ± SE; 40.2 ± 1.9%; n = 14; max = 180 kg) of body mass and 65.2 ± 2.6% (max = 80.6%) of storage energy between the fall prior to denning and the subsequent spring.

Females accompanied by cubs exhibited lower body mass (17.9 ± 3.0 kg less; F_3,356_ = 11.5, p < 0.0001; Bonferroni post-hoc tests: p < 0.001), calculated mass (14.7 ± 4.8 kg less; F_3,351_ = 3.58, p = 0.01), girth (3.7 ± 1.1 cm less; F_3,358_ = 10.8, p = 0.001) and CIs (BMI: 4.6 ± 0.7 kg/m^2^ less; F_3,357_ = 13.2, p < 0.0001; BCI: 0.69 ± 0.11 less; F_3,357_ = 13.4, p < 0.0001; energy density: 4.1 ± 0.7 MJ/kg less; F_3,355_ = 13.4, p < 0.0001) compared to females observed with yearlings, two-year olds, or without dependent young, but there was no difference among these other reproductive classes.

No size or condition measures taken the prior spring or fall (at time t) were related to the litter mass of cubs a female produced (95%CI of all coefficients overlapped 0; S2 Table). FIs assigned to females the spring or fall prior to denning were also not related to cub litter mass when accounting for litter size and cub capture date effects (spring: F_2,22_ = 1.3, n = 27, p = 0.30; fall: F_2,10_ = 1.9, n = 15, p = 0.20).

Cub litter mass (at year t) was linearly related to CIs, body mass, storage energy, girth, and size measures taken for mothers at year t (95% CI of β did not overlap 0 and p ≤ 0.05; S2 Table; Fig 2). Population, litter size and capture date influenced cub litter mass and were therefore included in all models (model containing these 3 variables: log-likelihood = -627.9, R^2^ = 0.59, χ^2^ = 157.8, p < 0.0001). R^2^ (range: 0.60–0.66) and log-likelihood values (range: -595.1 to -587.6) were similar for models that included a size and condition measures. The model including maternal BCI had the highest R^2^ (0.66) but the other variables in the model (population, litter size and capture date) accounted for the majority of variation in litter mass. Maternal age was not influential when combined with size or condition metrics, but was significant in models of first-year cub litter mass when included with litter size, population, and capture date but without size or condition metrics (β = 0.57, R^2^ = 0.62, 95% CI = 0.29–0.86, n = 164, p < 0.001). The litter masses of cubs accompanying females with a FI of 1 or 2 were 10.1 ± 3.6 kg lighter (β ± SE) than litter masses of females with an FI of 4 or 5 (95% CI = 3.1–17.2, n = 169, p = 0.005), but litter masses of females with FI of 3 did not differ from the other two FI categories (95% CI = -1.7–12.6, n = 169, p = 0.14).

The same suite of size and condition metrics that were related to cub litter mass were also related the litter mass of yearlings captured with her in the spring (maternal condition and litter mass both measured at time t). R^2^ (range: 0.73–0.77) and log-likelihood values (range: -530 to -521.7) were similar among size and condition measures. However, the base model including population, capture date, and litter size accounted for much of the variation in litter mass (log-likelihood = -565.6, R^2^ = 0.72, χ^2^ = 149.4, p < 0.0001) Maternal age did not influence yearling litter mass when included alone or in combination with size and condition measures. Yearling litter masses were similar for females assigned FIs of 3 compared to those with FIs of 4 or 5 (p = 0.80) but were 26.1 ± 13.8 kg lighter for females assigned a FI of 1 or 2 (F_2,128_ = 6.3, n = 127, p = 0.002) when including capture date and litter size effects (p < 0.01).

**Fig 3:**
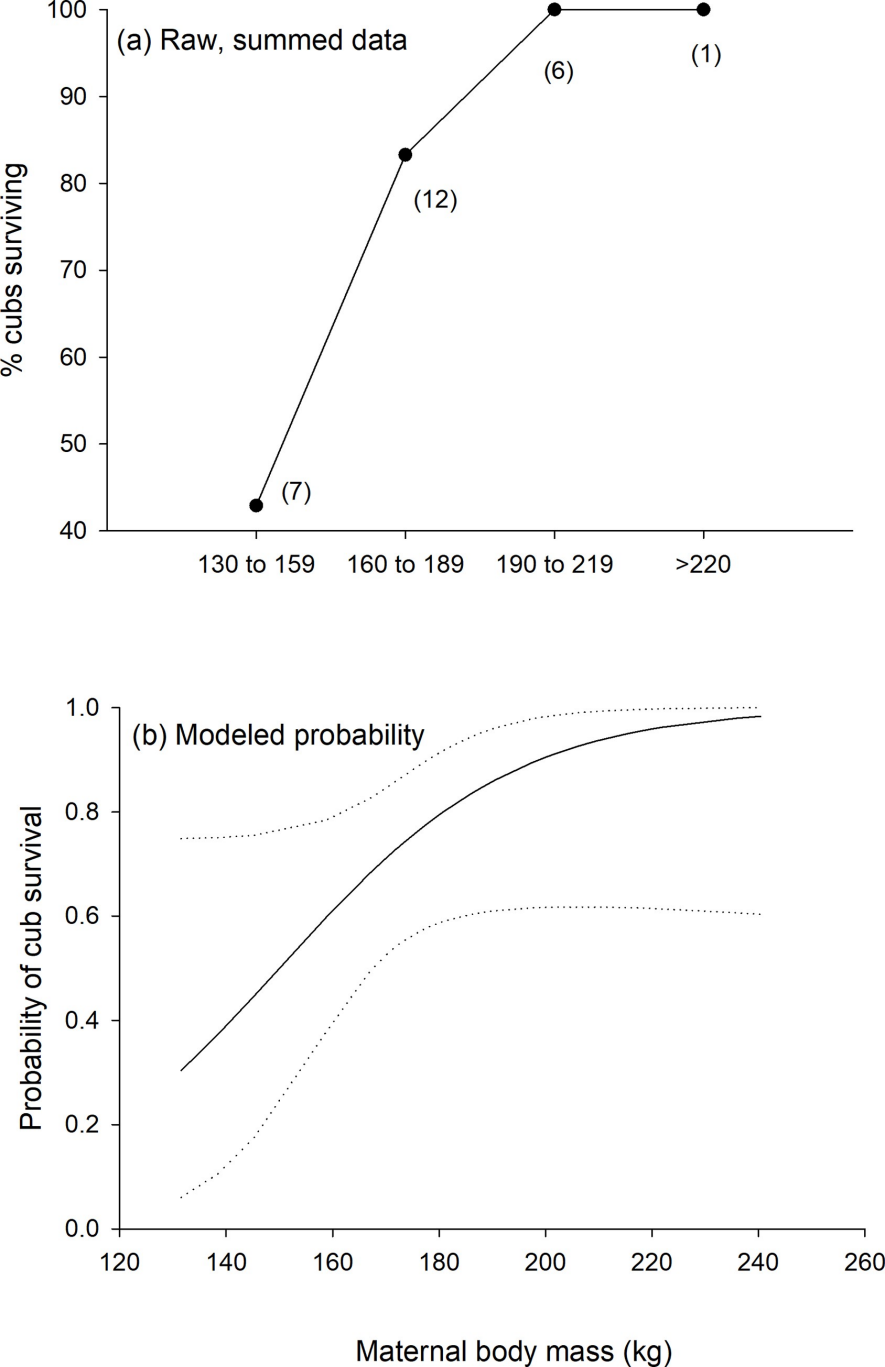
Relationship between the percent of cubs surviving and the mass of their mothers for raw data summarized among ranges of body mass (a) and for modeled probabilities (with 95% confidence intervals as dashed lines) based on a logistic regression (b). Cub survival was determined during the time between initial spring capture following den emergence to the following spring when their mothers were recaptured. Maternal mass was measured at initial capture of the cubs and was the only maternal metric related to cub survival.

Body mass was the only maternal size or condition metric that was related to cub survival during the year following spring emergence (Fig 3; log-likelihood = -19.1, β = 0.045, 95% CI = 0.002–0.089, n = 27, p = 0.05). Capture date, maternal age, and other maternal condition metrics did not influence cub survival, nor did cub survival differ between females with different FIs (95% CI = -0.64–3.11, p = 0.20).

### Cub body measurements and CIs related to survival

The probability that a cub survived between initial capture in the spring (year t) to the following spring (year t + 1) was related to the cub’s body length (log-likelihood = -18.5, β = 0.23, 95% CI = 0.08–0.38, n = 44, p < 0.001) and mass (log-likelihood = -19.0, β = 0.16, K = 2, 95% CI = 0.02–0.31, n = 73, p = 0.02). Cub survival (from year t to t + 1) was not related to capture date, cub sex, girth, skull width, calculated mass, BMI, BCI and storage energy measured at year t (i.e., 95% CI of β overlapped zero).

### Variation in CIs associated with age and length

**Fig 4:**
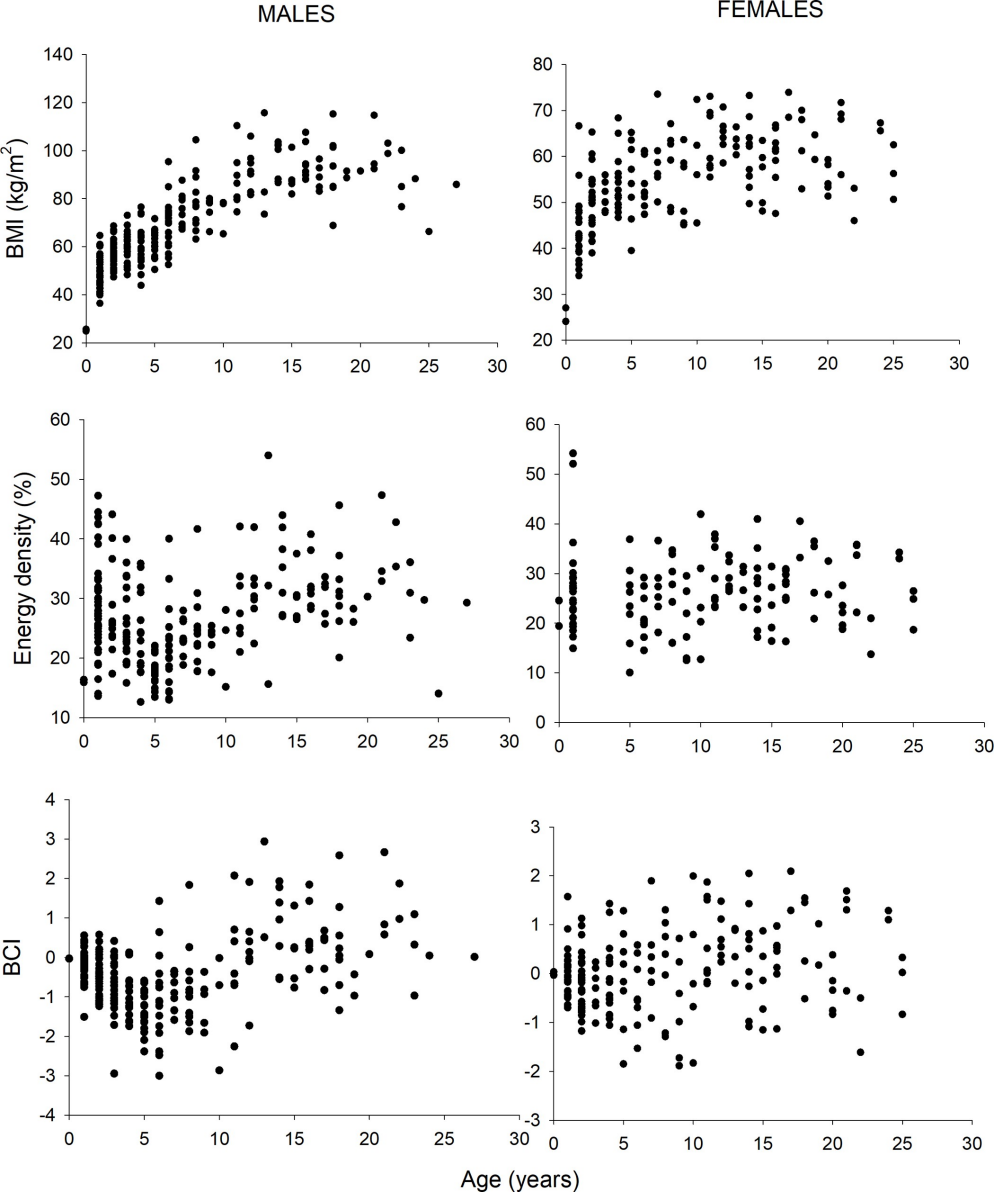
Values of three condition metrics (BCI, BMI, and energy density) relative to age for male and female polar bears captured in the Chukchi Sea.

**Fig 5:**
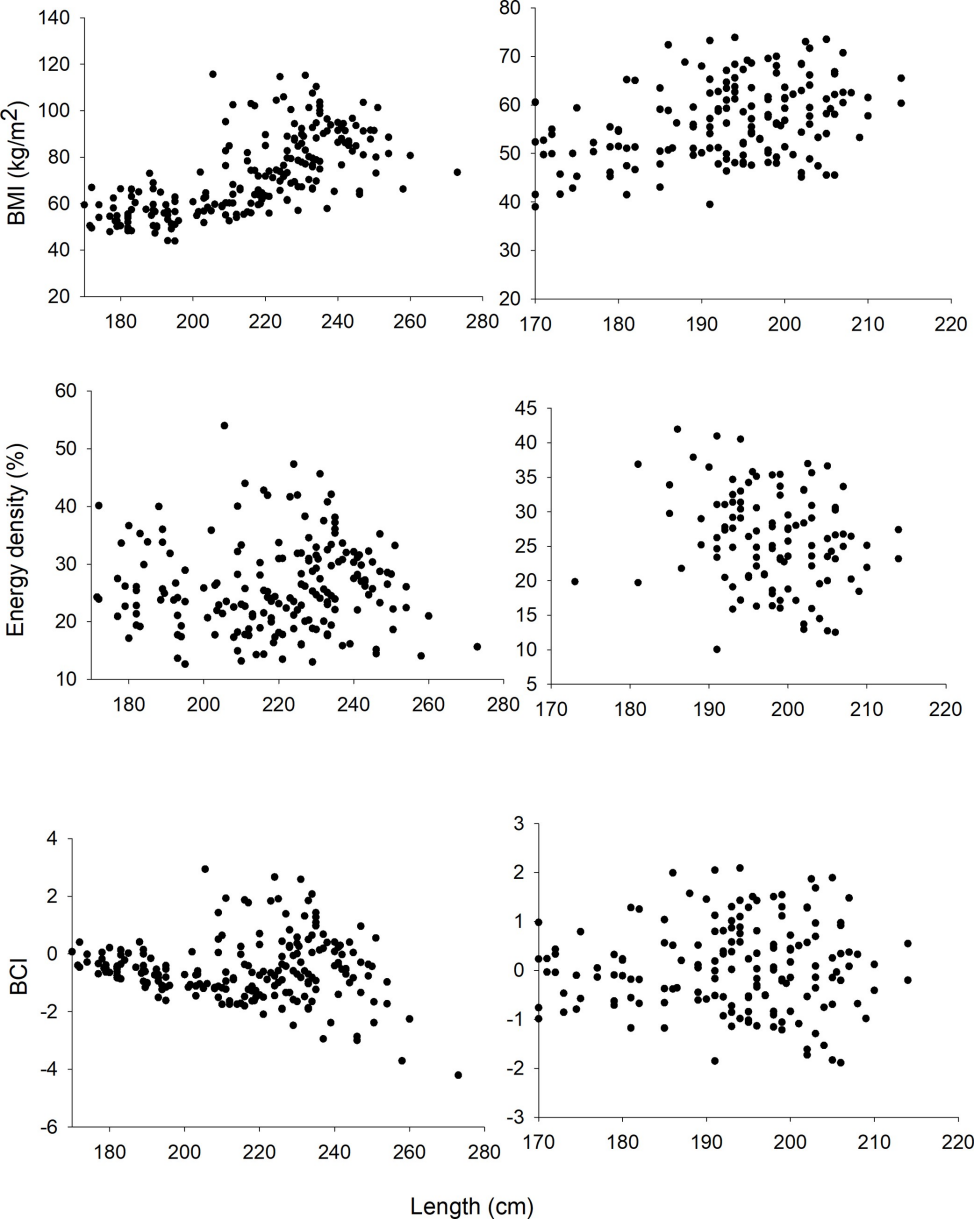
Values of three condition metrics (BCI, BMI, and energy density) relative to length for male and female polar bears captured in the Chukchi Sea. Data are shown for bears age 2 and older.

Measurement-based CIs (i.e. BMI, BCI, and energy density) were positively related to age for both growing and adult females and for growing males but not for energy density in adult females (Table 3; Fig 4). Measurement-based CIs were also negatively related to body length for adult males and females (Table 4; Fig 5). Some CIs exhibited positive or negative relationships to the length of yearlings and growing bears (Table 4; Fig 5). Only FIs of growing males, but not FIs for other sex/age groups, were related to age and length.

**Table 3:** Results of models examining whether condition indices are related to age for polar bears in four sex/age categories.

Measure	Age			
	Growing Females	Growing Males	Adult females	Adult males
	2–5 years	2–10 years	6+ years	11+ years
CONDITION INDICES				
BCI	**0.1 ± 0.0 (226)**	**0.04 ± 0.02 (392)**	**0.03 ± 0.009 (594)**	NS (192)
	***F***_***1*,*226***_ **= 4.6**	***F***_***1*,*392***_ **= 4.8**	***F***_***1*,*591***_ **= 4.8**	*F*_*1*,*189*_ = 0.06
	***p* = 0.03**	***p* = 0.03**	***p* = 0.03**	*p* = 0.8
BMI (kg/m^2^)	**2.0 ± 0.4 (221)**	**3.2 ± 0.2 (144)**	**0.2 ± 0.1 (593)**	NS (193)
	***F***_***1*,*218***_ **= 30.6**	***F***_***1*,*141***_ **= 8.4**	***F***_***1*,*590***_ **= 7.3**	*F*_*1*,*190*_ = 1.4
	***p* < 0.001**	***p* = 0.004**	***p* = 0.007**	*p* = 0.23
Energy Density (MJ/kg)	NA	**0.6 ± 4.8 (371)**	NS (592)	NS (192)
		***F***_***1*,*368***_ **= 22.9**	*F*_*1*,*589*_ = 0.5	*F*_*1*,*189*_ = 1.1
		***p* < 0.001**	*P* = 0.5	*p* = 0.3
Fatness index	NS (287)	**0.04 ± 0.01 (496)**	NS (565)	NS (307)
	*F*_*1*,*284*_ = 1.2	***F***_***1*,*493***_ **= 17.9**	*F*_*1*,*562*_ = 0.05	*F*_*1*,*304*_ = 1.3
	*p* = 0.28	***p* < 0.001**	*p* = 0.82	*p* = 0.26

A population variable (i.e., indicating whether a bear was from the Chukchi Sea or southern Beaufort Sea subpopulation) was included as a factor in all models. Adult categories were defined by the age at which growth in length is asymptotic. Bold text identifies significant relationships where coefficients (β –values) have standard errors that do not overlap 0. “NS” indicates no significant relationship. Sample sizes are in parentheses. Equations for estimating energy density of subadult females were not available.

**Table 4:** Results of linear regression models examining whether condition indices are related to body length for polar bears in four sex/age categories.

	Length				
	Yearlings	Growing Females	Growing Males	Adult females	Adult males
		2–5 years	2–10 years	6+ years	11+ years
CONDITION INDICES					
BCI	**0.012 ± 0.003 (188)**	NS (222)	**- 0.008 ± 0.002 (392)**	**-0.01 ± 0.005 (594)**	**-5.0± 0.6 (193)**
	***F***_***1*,*185***_ **= 11.8**	*F*_*1*,*219*_ = 2.5	***F***_***1*,*392***_ **= 11.6**	***F***_***1*,*591***_ **= 5.2**	***F***_***1*,*190***_ **= 25.7**
	***p* = 0.001**	*p* = 0.12	***p* = 0.001**	***p* = 0.02**	***p* < 0.0001**
BMI (kg/m^2^)	**0.7 ± 0.1 (189)**	**0.1 ± 0.0 (218)**	**1.1± 0.07 (384)**	**-0.11 ± 0.04 (593)**	**-5.0 ± 0.6 (193)**
	***F***_***1*,*186***_ **= 64.4, *p* < 0.001**	***F***_***1*,*218***_ **= 11.4, *p* = 0.001**	***F***_***1*,*381***_ **= 215.8, *p* = 0.004**	***F***_***1*,*590***_ **= 7.8, *p* = 0.005**	***F***_***1*,*190***_ **= 64.4, *p* < 0.001**
Energy Density (MJ/kg)	**-0.3 ± 0.0 (188)**	NA	NS (371)	**-0.40 ± 0.03 (592)**	**-0.9 ± 0.1 (193)**
	***F***_***1*,*185***_ **= 34.4, *p* < 0.001**		*F*_*1*,*398*_ = 0.35, *p* = 0.55	***F***_***1*,*589***_ **= 139.3, *p* < 0.001**	***F***_***1*,*190***_ **= 44.7, *p* <0.0001**
Fatness index	NS (219)	NS (268)	**0.005 ± 0.001 (449)**	NS (618)	NS (272)
	*F*_*1*,*215*_ = 3.5, *p* = 0.06	*F*_*1*,*266*_ = 0.2, *p* = 0.7	***F***_***1*,*446***_ **= 14.0, *p* < 0.001**	*F*_*1*,*615*_ = 3.2, *p* = 0.07	*F*_*1*,*270*_ = 2.4, *p* = 0.12

A population variable (i.e., indicating whether a bear was from the Chukchi Sea or southern Beaufort Sea subpopulation) was included as a factor in all models. Adult categories were defined by the age at which growth in length is asymptotic such that growing females and males are increasing in body length (see S2 Table) and adult females and males are increasing minimally or not at all in length. Bold text identifies significant relationships where coefficients (β –values) have standard errors that do not overlap 0. “NS” indicates no significant relationship. Sample sizes are in parentheses. Equations for estimating energy density of subadult females were not available.

Morphometric measurements, calculated body mass, and storage energy increased with age as would be expected for growing bears but also increased with age for adult females suggesting that females may continue to acquire body mass with age (S3 Table). The skull width of adult males increased with age but there was no pattern with age for length, girth, mass, calculated mass, or storage energy of adult males suggesting that this age grouping of males ≥ 11 years adequately identified males that had reached maximum size (S4 Table). Skull width, girth, mass, calculated mass, and storage energy were related to body length for bears of all sex and age groups demonstrating that these measures are affected by a bear’s structural size (S4 Table).

### Precision and accuracy of body measurements

For repeatedly captured males and females ≥11 years which should have achieved full body size, measurements of straight line length varied by up to 7.8 cm (mean difference between maximum and minimum measurement) on average for both sexes (n = 55 males and 43 females examined separately) and total body length varied by 6.5 and 6.1 cm, respectively for males (n = 27) and females (n = 18). The mean standard deviation for straight line lengths collected from the same individual was 5.7 cm (n = 20) and 4.2 cm (n = 13), respectively, for males and females whereas it was 3.9 cm (n = 27) and 4.0 cm (n = 11) for total body length. This represented 49.5% and 59.6% of the total standard deviation observed across individuals within the subpopulation for straight length (i.e., standard deviation in straight line length was 10.8 cm for males and 8.1 cm for females in the Beaufort Sea and 12.2 cm and 6 cm for CS) and 35.4% and 50.6% for total body length of males and females. There was no directional difference in length measures on the second capture compared to the first (paired t-tests had p > 0.10 for both length measures in males and females) that would suggest that the observed variation was a result of growth.

Bears that fed prior to capture had higher CIs compared to bears that had not fed for most sex and age classes captured in the Chukchi 2008–2017 (S5 Table). Body mass of adult females was the only size or condition measure that differed among bears that did and did not feed prior to capture (S5 Table).

An equation generated for CS bears that had not fed prior to capture (0.00007073girth^1.317^length^1.64^; R^2^ = 0.97; F_2,143_ = 2288.0, p < 0.0001) resulted in calculated mass 3.3 kg higher and variation of ±7.3 ± 6.1% compared to measured mass. Applying this same equation to CS bears that had fed prior to capture resulted in calculated mass values 12.5 kg lower and variation of ±9.9 ± 7.0%. compared to measured mass. The relationship between measured and calculated mass differed depending on gut fill (F_1,238_ = 5.07, p = 0.025) with no difference between sexes (p = 0.19; S1 Fig).

## Discussion

Metrics that combined body size and fatness, such as body mass, were more consistent predictors of fitness than CIs that standardized condition relative to structural size (Table 5).

**Table 5:** Summary of relationships between structural size and condition and female reproductive success and cub survival.

	Spring measure in year t related to cub production at year t + 1	Fall measure in year t related to cub production at year t + 1	Spring or fall measure in year t related to litter mass at year t + 1	Spring maternal measure related to first year cub litter mass at time t	Spring maternal measure related to yearling litter mass at time t	Maternal measure related to cub survival	Cub measure related to cub survival
STRUCTURAL SIZE							
Length				x	x		x
STRUCTURAL SIZE AND CONDITION							
Skull width				x	x		
Girth				x	x		
Scale body mass	x	x		x	x	x	x
Calculated body mass	x			x	x		
Storage Energy		x		x	x		
CONDITION INDICES							
BMI		x		x	x		
BCI		x		x	x		
Energy Density		x		x	x		

“x” indicates that the size or condition measure had a 95% confidence interval on β-value that did not overlap 0 and a p-value ≤ 0.05.

Below we discuss several reasons that CIs may not perform better than body mass in relating to fitness outcomes, including effects of structural body size on fitness, that CIs accumulate error associated with multiple morphometric measurements, and that they may tend to be lower for longer animals, particularly in younger, growing bears. We also note, and discuss further below, that age did not appear to be the underlying factor driving the relationships observed between body mass and fitness. Models including mass alone were better predictors than models that included age alone or in combination with mass or other CIs. Thus, the relationships observed between body mass, reproductive success and cub survival appear to be driven more by advantages associated with larger body mass than age-associated experience. We discuss these points further below.

### CIs as indicators of fitness

CIs of females performed well in predicting cub production when measured the fall prior to denning, but overall did not provide additional insights about fitness that were not already observed from body mass. Further, only body mass was related to reproductive outcomes when measured in the spring prior to denning. None of the size or CI measures taken in the spring or fall (year t) predicted the litter mass of the female measured after den emergence (t + 1) which may be a result of small sample sizes. Fall maternal mass has been linked to litter mass the spring after denning in Hudson Bay [84]. Thus, the lack of relationships in our study may be a result of smaller sample sizes.

One reason that CIs may not perform better than body mass alone is that both mass and length measurements have associated error that is cumulative when the two measures are combined [19, 58]. Bears identified as having recently fed had body masses that were 18.8 ± 8.4 kg greater for adult females and CIs that were consistently higher across most sex and age classes compared to those that were identified as having empty guts (S5 Table). Variation in repeated body length measurements of individual adult bears represented up to 60% of the variation observed across individuals within the two subpopulations. Other studies have similarly identified low precision of length measures [28, 80, 89, Thus, use of a single measurement to assess condition may reduce cumulative error which may enable improved detection of trends with fitness or other variables (i.e. time, environmental measures)].

CIs were also related to both age and body length for most sex and age classes (Tables 3 & 4). Older bears within age groups had higher CIs whereas length effects were primarily negative within age groups (Table 4). Thus, longer bears had lower CIs yet longer adult females had larger litter masses (Table 5) and longer cubs had higher rates of survival. Male and female polar bears continue to acquire body mass after the age of sexual maturity [5, 57, 84, this study] and after growth in length has become asymptotic [5]. This pattern suggests that young bears may initially invest more in increasing structural size and then increasingly accumulate energy reserves as they continue to age. Thus, relationships between age, length, and CIs are complex (see Figs 3 & 4) and it can be difficult to tease apart meaningful variation in CIs (i.e., that represent true variation in condition) versus those that result from changing relationships with length and age. Variation in the allometric relationship between mass and length is a pattern similarly documented in other species [84], that can result in inconsistencies in condition metrics applied across age groups.

Numerous studies have documented higher growth rates in mammals with increased nutritional intake [85, 86, Table 1 in 87] and corresponding higher juvenile survival [31, 46, 85, 88], reproductive success [89, 90], and survival rates of larger individuals [91] which suggests there is a benefit to a younger animal investing in structural size. Similarly, in our study, cub survival was associated with measures that reflect overall size (i.e. length and body mass) rather than energy reserves alone. Thus, lower CIs observed in longer bears may not accurately reflect the potential fitness advantages associated with size. Further, the traditional approach of separating animals into groups based on the age of sexual maturity is problematic for species that continue increasing size after sexual maturity.

FIs offer an appealing alternative to measurement-based condition because they can be applied in a variety of settings with no equipment (e.g., aerial surveys, harvest records, etc.) [20, 76]. Although FIs would seemingly be free of measurement error, growing males and females and adult females in this study that had not fed prior to capture were assigned lower FIs than those identified as having recently fed (S5 Table). Thus, FIs are not completely free of the issues discussed above for measurement-based approaches to assessing condition. Despite the effects of ingesta, FIs did reflect differences in litter mass of cubs and yearlings that accompanied females when compared among the lower (i.e., 1s & 2s) and higher scores (3s & 4s) but no other fitness indices varied among FIs. A recent study similarly documented relationships between FIs of adult females and the size of their litters [76]. These results suggest that although FIs are a subjective measure of condition, they can detect large variation that likely affects reproductive success.

### Role of body size in affecting fitness

Some aspects of fitness appear to be associated with body size. As a result, the standardization of CIs relative to length may affect their relationship with fitness outcomes.

In this study, the litter mass of cubs and yearlings accompanying a female was related to her body length suggesting a potential advantage of larger body size which could be genetic [90], environmental or both. Females of larger structural size may have a greater capacity to store energy required to support lactation [92], obtain larger prey [51], and be competitive at defending carcasses [suggested by 9] which aids in supporting cubs after den emergence. In a recent study across four Alaskan brown bear populations, body mass, length and skull size, but not BMI varied across adult females with the largest litter sizes occurring in areas where females were largest [93].

Investing energy reserves to maximize structural size may improve fitness for a variety of reasons, including increasing competitive ability for prey and mates and increasing fasting endurance. Large individuals metabolize energy reserves at a lower rate relative to body size such that per unit of storage, larger animals can survive longer periods of reduced food availability [94]. Although fasting endurance depends on proportional energy reserves at the start of a fast, a larger bear can withstand a longer period without food before body fat is reduced to the point of reproductive failure and death by starvation [12]. Further, larger body mass, rather than energy storage relative to body length, likely provides fitness advantages via effects on thermoregulation [94] and predation success [95]. That young, growing animals may invest additional resources towards growth at the expense of condition when food resources are abundant complicates the application of CIs that assess energy reserves relative to structural size. Rather it may be the combination of size and energy reserves that are important in affecting both short-term and long-term fitness of growing bears.

Our study adds to the growing body of evidence demonstrating that measures that combine fatness, skeletal muscle, and structural size, such as body mass, rather than proportional energy reserves or fatness alone (CIs), may best reflect variation in fitness. Body mass has been documented to be a better predictor of fitness than CIs in a variety of other species, including moose (*Alces* alces) [27], mule deer (*Odocoileus* hemionus) [30], roe deer (*Capreolus capreolus*) [29], elk (*Cervus elaphus*) [85], small mammals [26] and birds [11, 25]. In bears, larger body mass and structural size have been associated with greater prey availability, reproductive success, and cub survival [47, 48, 51, 96–98].

### Improving precision and accuracy of measurements

There were sources of error with three of the primary measures used directly or incorporated into CI calculations to track body condition in polar bears (i.e., measured and calculated body mass and length). Thus, how might these be addressed? Our result that calculated mass did not differ between bears identified with empty or full guts provides support that calculated mass is less sensitive to the amount of ingesta. However, calculated mass underestimated or overestimated scale body mass by up to 7% for individual bears that had not fed (and thereby did not have scale body masses biased by ingesta) and in doing so adds random variation within a data set [80, this study]. Further, studies have shown that equations for calculating body mass have to be generated for the population of interest and regularly updated using sample sizes of ≥150 weighed bears due to changes over time [49, 56]. The generation of separate equations for different subpopulations and time periods precludes comparisons of body mass over time and space. Some studies have identified relationships between mass calculated from girth measures and measures of female reproductive success [46, 93]. Using girth rather than both girth and length to calculate mass may be effective at reducing cumulative error. Some authors have avoided the issue of ingesta affecting measured mass by excluding bears that fed prior to capture [37] or capturing bears when they are fasting onshore. The methods used to estimate ingesta in our study were sufficient to detect meaningful differences in body mass and therefore may similarly be useful as a covariate in long-term monitoring. Although scale mass was affected by ingesta for adult females, it was still the most consistent predictor of fitness outcomes and there was no difference in scale body mass for other sex and age classes due to ingesta (S5 Table).

Of the two length measures examined in this study, total body length (measured along the curvature of the spine while a bear is on its side) was more precisely measured during repeated captures of individual bears than straight-line body length (measured as a straight line above the bear in sternal recumbency). Whether this results from the positioning of the bear or that the measurement can be made directly along the surface of the bear (rather than above the bear) is unclear. Additional research investigating relationships between fitness indices that use total body length or other length measures (i.e. along the curvature of the body with the bear sternally recumbent) could provide further insights for improving precision.

## Conclusions

CIs that attempt to standardize across bears varying in structural size were indicative of some aspects of female reproductive success [99, this study], whereas body mass, which is not standardized for structural size, was related to a broader suite of fitness metrics. Body mass, but not CIs, predicted cub production when measured during the spring a year prior to den emergence and the probability that a female’s cubs would survive the year following den emergence. Further, CIs were lower for longer bears despite longer adult females having larger litter masses and longer cubs having higher probabilities of survival. CIs may not be suitable indicators of fitness in growing bears because they do not reflect apparent advantages afforded by larger body size. The poorer performance of CIs as predictors of fitness may also be a result of cumulative error associated with imprecision in both mass and length measures. Our results, consistent with a number of studies of other species [11, 25–27, 29–31] suggest that body mass, rather than CIs [23], may be one of the most useful measures for linking nutritional changes to population dynamics.

## Supporting information

S1 Table Body size and condition metrics of females collected during the spring or fall prior to denning that exhibited relationships cub production based on logistic regression models. “Cub production” incorporates both whether a female produced cubs and whether they survived to be observed with her between March and early May. Size or condition metrics presented here had 95% confidence intervals on the coefficient (*β* –value) that did not overlap zero indicating it was an influential predictor variable. Linear and non-linear (i.e. quadratic terms) relationships between size and condition metrics and cub production were examined. As indicated in Table 2, maternal age and cub capture date were included in models with size and condition metrics when the 95% confidence interval on the coefficient (*β* –value) did not overlap zero.
(DOCX)

S2 Table Results of linear models examining relationships between adult female size and condition measures and the mass of her litter of first year cubs or yearlings. Size and condition measures of the female were collected either the spring (“prior spring”) or fall (“prior fall”) prior to her subsequent capture with a litter or simultaneous to measurement of litter mass during the spring (i.e. “at spring capture”). Linear and non-linear relationships (i.e. as quadratic terms) between size and condition measures and litter mass were considered. The date in which the family group was captured (cdate), the number of cubs (litsize) in the litter, and maternal age (age) were included as covariates metric if the 95% confidence interval on the coefficient (*β* –value) did not overlap zero. “SB” and “CS” indicate the subpopulation data used in the analysis. A covariate for subpopulation (pop) was included in candidate models for data sets where data from both subpopulations were used (subpopulation data used indicated as “SB” for southern Beaufort and “CS” for Chukchi Sea”). This table presents results for models in which the size or condition metric had a 95% confidence interval on the coefficient (*β* –value) that did not overlap zero indicating it was an influential predictor variable. Where size and condition measures were influential in multiple models with and without covariates or as a linear and non-linear parameter, the model with the higher R^2^ and lowest log-likelihood are presented.
(DOCX)

S3 Table Results of models examining the effects of age on morphometric measures and calculated measures of body mass and storage energy for polar bears in four sex/age categories. A population variable (i.e., indicating whether a bear was from the Chukchi Sea or southern Beaufort Sea subpopulation) was included as a factor in all models. Adult categories were defined by the age at which growth in length is asymptotic. Adult categories were defined by the age at which growth in length and mass is asymptotic. Bold text identifies significant relationships where coefficients (β –values) have standard errors that do not overlap 0. “NS” indicates no significant relationship. Sample sizes are in parentheses. Equations for estimating storage energy of subadult females were not available.
(DOCX)

S4 Table Results of models examining the effects of length on morphometric measures and calculated measures of body mass and storage energy for polar bears in four sex/age categories. Adult categories were defined by the age at which growth in length is asymptotic. Bold text identifies significant relationships where coefficients (β –values) have standard errors that do not overlap 0. “NS” indicates no significant relationship. Sample sizes are in parentheses. Equations for estimating storage energy of subadult females were not available.
(DOCX)

S5 Table Results of ANCOVAs comparing morphometric measures and CIs between polar bears of different sex and age classes that were identified as having fed just prior to measurement (i.e., F = full) with those that were identified as being empty (E). “NS” (i.e., “not significant”) indicates that the measure did not differ between empty and full bears. “E<F” indicates full bears had a higher measure than empty bears and “E>F” indicates empty bears had a higher measure than full bears. Significant differences are shown in bold. Coefficients (β –values) are provided with standard errors. Capture date and age effects were included in ANCOVAs for growing and adult males and females if *p* ≤ 0.05. ANCOVAs for yearlings included a sex effect and capture date.
(DOCX)

S1 Fig Relationship between calculated (CBM) and scale body mass (scale mass) for polar bears captured in the Chukchi Sea that were identified as having fed recently (dotted line) or having not fed recently (dashed line) based on direct observations of feeding behavior and palpitation to estimate gut content. The black solid line represents a 1:1 relationship.
(DOCX)
