# Supplementary material for: Identifying reliable indicators of fitness in polar bears
Source: PLoS One. 2020 Aug 19;15(8):e0237444. doi: 10.1371/journal.pone.0237444 (PMC7437918; doi:10.1371/journal.pone.0237444)
Supplement: S1 Table — “Cub production” incorporates both whether a female produced cubs and whether they survived to be observed with her between March and early May. Size or condition metrics presented here had 95% confidence intervals on the coefficient (β –value) that did not overlap zero indicating it was an influential predictor variable. Linear and non-linear (i.e. quadratic terms) relationships between size and condition metrics and cub production were examined. As indicated in Table 2, maternal age and cub capture date were included in models with size and condition metrics when the 95% confidence interval on the coefficient (β –value) did not overlap zero. (DOCX) [file pone.0237444.s001.docx]

**S1 Table. Body size and condition metrics of females collected during the spring or fall prior to denning that exhibited relationships cub production based on logistic regression models.**  “Cub production” incorporates both whether a female produced cubs and whether they survived to be observed with her between March and early May. Size or condition metrics presented here had 95% confidence intervals on the coefficient (*β* – value) that did not overlap zero indicating it was an influential predictor variable. Linear and non-linear (i.e. quadratic terms) relationships between size and condition metrics and cub production were examined. As indicated in Table 2, maternal age and cub capture date were included in models with size and condition metrics when the 95% confidence interval on the coefficient (*β* – value) did not overlap zero.

| Model | Log L | Metric *β* (95% CI) | Metric  p-value |
| --- | --- | --- | --- |

| CUB PRODUCTION (Related to maternal condition the prior spring); n = 37 | | | |  |
| --- | --- | --- | --- | --- |
| mass | -16.9 | 0.06 (0.02, 0.10) | 0.008 | |
| Mass^2^ | -17.6 | 0.00012 (0.00002, 0.00015) | 0.014 | |
| Calculated mass | -21.8 | 0.03 (0.0004, 0.06) | 0.05 | |
| CUB PRODUCTION (Related to maternal condition the prior fall); n = 20 | | | |  |
| energy density | -5.0 | 0.32 (0.001, 0.650) | 0.05 | |
| energy density^2^ | -5.0 | 0.005 (0.0002, 0.011) | 0.06 | |
| BMI | -5.2 | 0.28 (0.001, 0.055) | 0.003 | |
| BMI^2^ | -5.2 | 0.002 (7 x 10^-7^, 0.004) | 0.05 | |
| mass | -5.4 | 0.05 (0.01, 0.12) | 0.02 | |
| BCI | -5.4 | 1.79 (0.03, 3.56) | 0.05 | |
| BCI^2^ | -5.5 | 0.24 (0.004, 0.476) | 0.05 | |
| Storage energy | -5.6 | 0.003 (0.00001, 0.005) | 0.05 | |
| Storage energy^2^ | -5.7 | 3.4 x 10^-7^  (3.3 x 10^-9^, 6.8 x 10^-7^) | 0.05 | |
