# Supplementary material for: Identifying reliable indicators of fitness in polar bears
Source: PLoS One. 2020 Aug 19;15(8):e0237444. doi: 10.1371/journal.pone.0237444 (PMC7437918; doi:10.1371/journal.pone.0237444)
Supplement: S2 Table — Size and condition measures of the female were collected either the spring (“prior spring”) or fall (“prior fall”) prior to her subsequent capture with a litter or simultaneous to measurement of litter mass during the spring (i.e. “at spring capture”). Linear and non-linear relationships (i.e. as quadratic terms) between size and condition measures and litter mass were considered. The date in which the family group was captured (cdate), the number of cubs (litsize) in the litter, and maternal age (age) were included as covariates metric if the 95% confidence interval on the coefficient (β –value) did not overlap zero. “SB” and “CS” indicate the subpopulation data used in the analysis. A covariate for subpopulation (pop) was included in candidate models for data sets where data from both subpopulations were used (subpopulation data used indicated as “SB” for southern Beaufort and “CS” for Chukchi Sea”). This table presents results for models in which the size or condition metric had a 95% confidence interval on the coefficient (β –value) that did not overlap zero indicating it was an influential predictor variable. Where size and condition measures were influential in multiple models with and without covariates or as a linear and non-linear parameter, the model with the higher R2 and lowest log-likelihood are presented. (DOCX) [file pone.0237444.s002.docx]

**S2 Table.** **Results of linear models examining relationships between adult female size and condition measures and the mass of her litter of first year cubs or yearlings.** Size and condition measures of the female were collected either the spring (“prior spring”) or fall (“prior fall”) prior to her subsequent capture with a litter or simultaneous to measurement of litter mass during the spring (i.e. “at spring capture”). Linear and non-linear relationships (i.e. as quadratic terms) between size and condition measures and litter mass were considered. The date in which the family group was captured (cdate), the number of cubs (litsize) in the litter, and maternal age (age) were included as covariates metric if the 95% confidence interval on the coefficient (*β* – value) did not overlap zero. “SB” and “CS” indicate the subpopulation data used in the analysis. A covariate for subpopulation (pop) was included in candidate models for data sets where data from both subpopulations were used (subpopulation data used indicated as “SB” for southern Beaufort and “CS” for Chukchi Sea”). This table presents results for models in which the size or condition metric had a 95% confidence interval on the coefficient (*β* – value) that did not overlap zero indicating it was an influential predictor variable. Where size and condition measures were influential in multiple models with and without covariates or as a linear and non-linear parameter, the model with the higher R^2^ and lowest log-likelihood are presented.

|  | Log L | R^2^ | Metric  Β (95% CI) | Metric  p-value |
| --- | --- | --- | --- | --- |
| FIRST YEAR CUB LITTER MASS (related to maternal condition the prior spring) – SB only n = 18 | | | | |
| Energy density + cdate + litsize | -61.2 | 0.36 | 0.5 (-1.5, 0.6) | 0.37 |
| FIRST YEAR CUB LITTER MASS (Related to maternal condition the prior fall) – SB only n = 19 | | | | |
| cdate + litsize | -47.6 | 0.56 | NA | NA |
| FIRST YEAR CUB LITTER MASS AT SPRING CAPTURE – SB + CS; All models below included litter size, population, and capture date; n = 164 | | | | |
| BCI | -582.6 | 0.66 | 4.7 (3.1, 6.3) | < 0.0001 |
| Girth (cm) | -585.3 | 0.63 | 0.44 (0.28, 0.61) | < 0.0001 |
| Mass (kg) | -587.5 | 0.63 | 0.14 (0.08, 0.19) | < 0.0001 |
| Age (years) | -590.4 | 0.63 | 0.57 (0.29, 0.86) | < 0.0001 |
| Fatness index | -589.3 | NA | See main text | <0.001 |
| BMI (kg/m^2^) | -590.7 | 0.64 | 0.51 (0.25, 0.76) | < 0.0001 |
| Storage energy (MJ) | -590.7 | 0.64 | 0.005  (0.002, 0.008) | < 0.0001 |
| Energy density (MJ/kg) | -591.7 | 0.64 | 0.48 (0.212, 0.74) | <0.0001 |
| Skull width (cm) | -594.6 | 0.60 | 1.9 (0.4, 3.3) | 0.01 |
| Calculated Mass (kg) | -595.1 | 0.63 | 0.10 (0.06, 0.14) | < 0.0001 |
| Length (cm) | -595.9 | 0.62 | 0.162 (0.001, 0.323) | 0.05 |
| YEARLING LITTER MASS (Maternal condition when litter mass was measured)  SB + CS; All models below included litter size, population, and capture date; n = 107 | | | | |
|  | Log L | R^2^ | Condition metric  β ± SE | Condition metric  p-value |
| Girth | -521.7 | 0.77 | 1.4 (0.8, 1.9) | < 0.0001 |
| Calculated mass | -522.0 | 0.77 | 0.3 (0.2, 0.5) | < 0.0001 |
| Mass | -524.5 | 0.77 | 0.51 (0.27, 0.75) | < 0.0001 |
| Storage energy | -526.9 | 0.75 | 0.018 (0.007, 0.028) | 0.001 |
| BMI | -527.8 | 0.75 | 1.6 (0.6, 2.7) | 0.002 |
| BCI | -529.2 | 0.73 | 10.1 (2.3, 17.8) | 0.01 |
| Energy Density | -529.8 | 0.73 | 1.3 (0.2, 2.4) | 0.02 |
| Skull width | -530.0 | 0.73 | 7.1 (0.8, 13.5) | 0.03 |
| Length | -553.5 | 0.73 | 1.2 (0.14, 2.28) | 0.03 |
