# Supplementary material for: Identifying reliable indicators of fitness in polar bears
Source: PLoS One. 2020 Aug 19;15(8):e0237444. doi: 10.1371/journal.pone.0237444 (PMC7437918; doi:10.1371/journal.pone.0237444)
Supplement: S3 Table — A population variable (i.e., indicating whether a bear was from the Chukchi Sea or southern Beaufort Sea subpopulation) was included as a factor in all models. Adult categories were defined by the age at which growth in length is asymptotic. Adult categories were defined by the age at which growth in length and mass is asymptotic. Bold text identifies significant relationships where coefficients (β –values) have standard errors that do not overlap 0. “NS” indicates no significant relationship. Sample sizes are in parentheses. Equations for estimating storage energy of subadult females were not available. (DOCX) [file pone.0237444.s003.docx]

**S3 Table.** **Results of models examining the effects of age on morphometric measures and calculated measures of body mass and storage energy for polar bears in four sex/age categories.** A population variable (i.e., indicating whether a bear was from the Chukchi Sea or southern Beaufort Sea subpopulation) was included as a factor in all models. Adult categories were defined by the age at which growth in length is asymptotic. Adult categories were defined by the age at which growth in length and mass is asymptotic. Bold text identifies significant relationships where coefficients (β –values) have standard errors that do not overlap 0. “NS” indicates no significant relationship. Sample sizes are in parentheses. Equations for estimating storage energy of subadult females were not available.

| **Measure** | **Age** | | | |
| --- | --- | --- | --- | --- |
|  | Growing Females  2-5 years | Growing Males  2-10 years | Adult females  6+ years | Adult males  11+ years |
| DIRECT BODY MEASUREMENTS | | | | |
| Length (cm) | **7.1 ± 0.5 (305)**  ***F_1,302_* = 170.2**  ***p* < 0.001** | **6.2 ± 0.3 (456)**  ***F_1,453_* =593.3**  ***p* < 0.001** | **0.38 ± 0.07 (596)**  ***F_1,593_* = 31.3**  ***p* < 0.001** | NS (275)  *F_1,275_* = 0.1  *p* =0.75 |
| Skull Width (cm) | **0.8 ± 0.1 (297)**  ***F_1,297_* = 215.5**  ***p* < 0.001** | **0.9 ± 0.0 (487)**  ***F_1,485_* =947.9**  ***p* < 0.001** | **0.08 ± 0.008 (602)**  **F_1,599_ = 116.4, P < 0.001** | **0.09 ± 0.02 (298)**  ***F_1,295_* = 17.9**  ***p* < 0.001** |
| Girth (cm) | **5.2 ± 0.5 (322)**  ***F_1,319_* = 97.3**  ***p* < 0.0001** | **5.1 ± 0.2 (494)**  ***F_1,491_* = 460.0**  ***p* < 0.001** | **0.39 ± 0.08 (605)**  ***F_1,602_* = 22.7**  ***p* < 0.001** | NS (303)  *F_1,300_*= 3.2  *p* = 0.08 |
| Mass (kg) | **17.1 ± 1.4 (237)**  ***F_1,237_* = 141.0**  ***p* < 0.001** | **29.7 ± 1.1 (434)**  ***F_1,431_* = 747.4**  ***p* < 0.001** | **1.4 ± 0.3 (604)**  ***F_1,601_* = 26.8**  ***p* < 0.001** | NS (213)  *F_1,210_* = 1.0  *p* =0.31 |
| CALCULATED METRICS COMBINING STRUCTURAL SIZE AND CONDITION | | | | |
| Calculated Mass (kg) | **21.3 ± 1.7 (278)**  ***F_1,275_* = 150.8**  ***p* < 0.001** | **27.1 ± 1.6 (496)**  ***F_1,493_* = 283.8**  ***p* < 0.001** | **1.4 ± 0.4 (461)**  ***F_1,458_* = 13.2**  ***p* < 0.001** | NS (214)  *F_1,211_* = 1.1  *p* =0.29 |
| Storage Energy (MJ) | NA | **287.6 ± 22.6 (398)**  ***F_1,394_* = 162.6**  ***p* <0.001** | **19.8 ± 6.3 (592)**  ***F_1,589_* = 9.9**  ***p* = 0.002** | NS (192)  *F_1,189_* = 0.4  *p* =0.6 |
