# Supplementary material for: Identifying reliable indicators of fitness in polar bears
Source: PLoS One. 2020 Aug 19;15(8):e0237444. doi: 10.1371/journal.pone.0237444 (PMC7437918; doi:10.1371/journal.pone.0237444)
Supplement: S5 Table — “NS” (i.e., “not significant”) indicates that the measure did not differ between empty and full bears. “E<F” indicates full bears had a higher measure than empty bears and “E>F” indicates empty bears had a higher measure than full bears. Significant differences are shown in bold. Coefficients (β –values) are provided with standard errors. Capture date and age effects were included in ANCOVAs for growing and adult males and females if p ≤ 0.05. ANCOVAs for yearlings included a sex effect and capture date. (DOCX) [file pone.0237444.s005.docx]

**S5 Table.** **Results of ANCOVAs comparing morphometric measures and CIs between polar bears of different sex and age classes that were identified as having fed just prior to measurement (i.e., F = full) with those that were identified as being empty (E).** “NS” (i.e., “not significant”) indicates that the measure did not differ between empty and full bears. “E<F” indicates full bears had a higher measure than empty bears and “E>F” indicates empty bears had a higher measure than full bears. Significant differences are shown in bold. Coefficients (β – values) are provided with standard errors. Capture date and age effects were included in ANCOVAs for growing and adult males and females if *p* ≤ 0.05. ANCOVAs for yearlings included a sex effect and capture date.

|  | Yearlings | Growing females 2-5 years | Growing males 2-10 years | Females ≥6 years | Males  ≥11 years |
| --- | --- | --- | --- | --- | --- |
| Scale body mass (kg) | NS (62)  *F_1,59_* = 0.1  *p* = 0.71 | NS (31)  *F_1,28_* = 0.08, *p* =0.78 | NS (89)  *F_1,86_* = 0.90, *p* =0.35 | **E<F (58)**  **18.8 ± 8.4**  ***F_1,55_* = 5.0**  ***p* =0.03** | NS (56)  E<F  *F_1,53_* = 3.4  *p* = 0.07 |
| Calculated body mass (kg) | NS (57)  *F_1,54_* = 1.5  *p* = 0.23 | NS (30)  *F_1,27_*= 0.0  *p* =0.99 | NS (88)  *F_1,85_* = 0.13, *p* =0.73 | NS (58)  *F_1,55_*= 0.04  *p* =0.85 | NS (56)  *F_1,53_*= 0.18  *p* =0.68 |
| Girth (cm) | NS (60)  *F_1,57_* = 0.8  *p* = 0.37 | NS (31)  *F_1,28_*= 0.0  *P =* 0.98 | NS (88)  *F_1,85_* = 1.6, *p* =0.20 | NS (60)  *F_1,57_*= 0.86  *p* =0.36 | NS (57)  *F_1,54_*= 2.3  *p* =0.13 |
| Body length (cm) | NS (57)  *F_1,54_* = 0.5  *p* = 0.48 | NS (30)  *F_1,27_*= 0.08, *p* =0.78 | NS (88)  *F_1,85_* = 0.6  *p* =0.4 | NS (58)  *F_1,55_*= 2.1  *p* =0.15 | NS (57)  *F_1,54_*= 0.5  *p* =0.49 |
| Skull width (cm) | NS (65)  *F_1,62_* = 2.5  *p* = 0.12 | NS (31)  *F_1,28_*= 3.6  *p* =0.07 | NS (90)  *F_1,87_* = 0.45, *p* =0.5 | NS (60)  *F_1,57_*= 0.00  *p* =0.98 | NS (61)  *F_1,58_*= 0.4  *p* =0.53 |
| Energy density (MJ/kg) | NS (57)  *F_1,54_* = 0.00  *p* = 0.96 | NA | **E<F (75)**  **4.1 ± 1.5**  ***F_1,72_* = 7.8 *p* =0.007** | **E<F (58)**  **5.1 ± 1.7**  ***F_1,55_* = 8.7**  ***p* = 0.005** | **E<F (55)**  **7.6 ± 0.008**  ***F_1,52_* = 7.6**  ***p* = 0.008** |
| BCI | NS (57)  *F_1,54_* = 0.28  *p* = 0.60 | NS (30)  *F_1,27_* = 0.00, *p* = 0.96 |  | **E<F (57)**  **0.65 ± 0.24**  ***F_1,54_* =2.9**  ***p* = 0.009** | **E<F (55)**  **1.0 ± 0.4**  ***F_1,52_* = 6.4**  ***p* = 0.01** |
| BMI (kg/m^2^) | NS (57)  *F_1,54_* = 0.02  *p* = 0.88 | NS (30)  *F_1,27_* = 0.00, *p* = 0.93 | **E<F (88)**  **4.1 ± 1.5**  ***F_1,85_* = 5.5, *p* =0.02** | **E<F (58)**  **7.0 ± 2.7**  ***F_1,55_* = 6.6**  ***p* = 0.01** | **E<F (55)**  **9.4 ± 3.2**  ***F_1,52_* = 8.9**  ***p* = 0.004** |
| Fatness index | NS (65)  *F_1,62_* = 0.97  *p* = 0.33 | **E<F (31)**  **+0.29 ± 0.14**  ***F_1,28_* = 4.4**  ***p* = 0.05** | **E<F (91)**  **0.26 ± 0.10**  ***F_1,88_* = 6.8, *p* =0.01** | **E<F (61)**  **+0.45 ± 0.12**  ***F_1,58_* = 14.0**  ***p* < 0.001** | NS (61)  *F_1,58_*= 1.2  *p* =0.27 |
