# Supplementary material for: Identifying reliable indicators of fitness in polar bears
Source: PLoS One. 2020 Aug 19;15(8):e0237444. doi: 10.1371/journal.pone.0237444 (PMC7437918; doi:10.1371/journal.pone.0237444)
Supplement: S1 Fig — The black solid line represents a 1:1 relationship. (DOCX) [file pone.0237444.s006.docx]

**
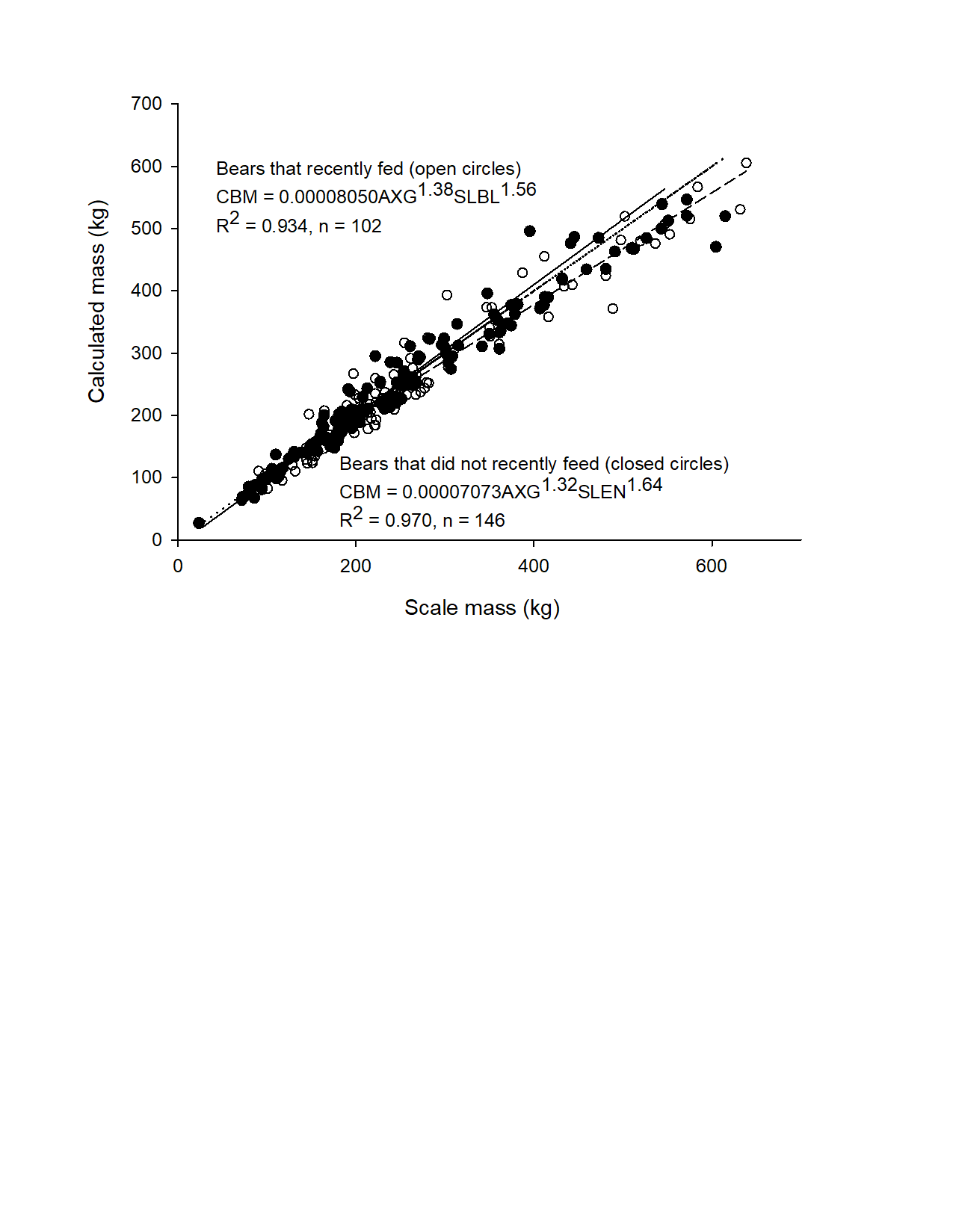
**

**S1 Fig.** **Relationship between calculated (CBM) and scale body mass (scale mass) for polar bears captured in the Chukchi Sea that were identified as having fed recently (dotted line) or having not fed recently (dashed line) based on direct observations of feeding behavior and palpitation to estimate gut content.** The black solid line represents a 1:1 relationship.
